# Comparison of usefulness of two tests measuring anaerobic performance of untrained and soccer-training girls U12

**DOI:** 10.1038/s41598-023-46825-2

**Published:** 2023-11-09

**Authors:** Agnieszka Danuta Jastrzębska

**Affiliations:** Department of Physiology and Biochemistry, Wroclaw University of Health and Sport Sciences, 51-612 Wrocław, Poland

**Keywords:** Developmental biology, Physiology

## Abstract

The study aimed to investigate the usefulness of the Running-based Anaerobic Sprint Test (RAST) in anaerobic performance estimation in trained and untrained girls U12, and the effect of an 8-week training period in female U12 soccer players on anaerobic performance. A comparative study of two structurally different anaerobic tests was performed to reach the goal. The study was designed as a non-randomized, controlled before-and-after trial. Fourteen female soccer players (FSP) and twelve untrained girls (UNT) participated in the study. During that time, all participants were subjected to school’s physical education classes and the FSP additionally participated in regular soccer training. The anaerobic performance was evaluated twice, within 8-weeks period, using the traditional Wingate test (WAnT) and the RAST. A significant increase in the anaerobic performance of the FSP was noted (p < 0.05). In both tests peak, average, and relative power were significantly improved (p < 0.005). Nevertheless, strong, statistically significant (p < 0.05) correlation coefficients (0.50 < r < 0.70) were found for pre-training measurements between the WAnT and the RAST power parameters in absolute values. In UNT group significant improvement was found in peak, average and min power (p < 0.05) in the RAST. The improvement in anaerobic performance after training along with the strong correlation noted between the WAnT and the RAST power parameters prove the usefulness of the RAST in assessing anaerobic capacity in female youth athletes. Its simplicity encourages its use in monitoring anaerobic capacity in both trained and untrained girls.

## Introduction

Soccer has been the most popular sport for decades, with the largest number of participants worldwide. Its popularity is increasing among children and teenagers, both girls and boys, however, scientific interest is mainly focused on the male population^1–4^. Both the aerobic^5–7^ and anaerobic^4,6,7^ performance of soccer competitors were widely examined.

By nature, soccer is a very irregular game. Even though over 90% of a game is covered by aerobic metabolism, soccer players need perform repeated short-duration (2–7 s), maximal or near maximal, multi-directional bouts throughout the game as these play a significant role in the crucial moments of the match^8^. The long-duration activity is interspersed with short, high-intensity bouts which require continuous ATP supply. The observation that top-class male and female soccer players perform 150–250 and 125–154 intense sprints during a match, respectively^9^, indicates a high rate of anaerobic energy turnover during periods of the match. Therefore, parallel with technical and tactical skills, strong emphasis should be placed on anaerobic skills. However, little information is available about the types of training given to the youngest competitors. In prior studies, researchers aimed to validate different regimens for interval^6,10–12^ or plyometric training^13–15^, however, the results are inconclusive. Lack of positive effects of 4-weeks of sprint interval training (SIT)^11^ in prepubescent boys, and 8-weeks of high intensity interval training (HIIT)^12^ in prepubescent girls were noticed. In contrast, 9 weeks of interval training applied to prepubescent boys increases the indices of anaerobic capacity^16^. Marzouki et al.^17^ reported that plyometric exercise, introduced to the usual physical education classes, performed with low to moderate-high intensity drills, twice-weekly for 4 weeks, improves the anaerobic fitness in children of both sexes U12. Bedoya et al.^15^ in the review paper claimed that in young soccer players also plyometric training can be applied, but only in relatively low volumes and frequencies and should be individualized. However, no data on plyometric training for female U12 soccer players are available. The HIIT was also proposed as a retraining form after detraining period^18^. Clemente et al.^18^ used HIIT and small sided games (SSG) in young soccer player boys in 4-weeks retraining period after 4-weeks detraining period. The authors concluded that both interventions improved the physical fitness of participants, however, the 4 weeks of retraining was not enough to return to baseline. The inconsistent results may indicate that the training impact variation in prepubescent children may depend on the applied training type, its duration and targeted population.. With maturity, the mentioned training types improve the anaerobic capacity indices in both sexes^11,15^ which is resulted by increases muscle mass^19^ and neural coordination^15^, although in boys the changes are more rapid and stronger^15,20^.

Training is one of the components of the process of shaping physical capacity. The second component is the possibility of assessing the input state before the intervention and estimating the effects of the applied intervention. Throughout the training process, it should be remembered that the tests evaluating the fitness level/adaptation process and the training loads should be selected adequately to the age/maturity and exercise capacity of the subjects. This ensures accurate assessment of fitness and effective development of capacity through exercise intervention.

Despite the extensive literature on various aspects of football^4,14,18,21^, there is a little information about women’s football^5,10,13^, especially about young girls’ football. Analysis of women’s and girls’ soccer is mainly confined to match analysis in elite senior players (competition)^9^. To the best of the author’s knowledge, no other study has assessed the anaerobic performance of female U-12 soccer players using two anaerobic tests for this purpose. One of the most widely used laboratory tests for assessing anaerobic performance is the WAnT, which is considered the gold standard. The WAnT is based on 30 s of cycling against a weight-based load with maximum intensity. As a laboratory tests it requires expensive equipment, trained personnel, and a dedicated laboratory space. Furthermore, cycling is less dependent on coordination skills, and cycling movement structure does not accurately reflect the movement patterns required in soccer, where the whole-body weight must be actively carried from one place to another. Corresponding to the WAnT, in recent years the Running-based Anaerobic Sprint Test (RAST) has gained popularity in assessing speed and anaerobic performance in football players. RAST is a simple field test for estimating anaerobic power capacity based on six 35 m sprints with a 10 s passive rest between^21^. Although it seems to be a simple form, it requires appropriate motor and coordination skills. Its low cost also should be considered, as for young amateurs and coaches, it increases its accessibility. Both the WAnT and the RAST are accepted as tools for assessing anaerobic capacity in soccer players^22–24^, including children^4^. Both the reliability and validity of the RAST^23,25,26^ and WAnT^27,28^ have been proved.

The research aimed to determine whether the running-based anaerobic sprint test can effectively estimate anaerobic performance in young girls, regardless of their training status or performance level. To confirm this, the study evaluated the relationship between performance variables obtained in the RAST and the WAnT tests in U12 untrained girls and soccer players in pre-season training. The correlation between the anaerobic capacity parameters obtained in these two tests, both before and after 8 weeks of soccer training, was used to assess the fitness of young female athletes and confirm the usefulness of the RAST for evaluating anaerobic capacity in girls' football training. Additionally, comparing the anaerobic performance of soccer-trained and untrained girls using these two structurally different tests may provide insight into the methods used for estimating anaerobic performance in young girls.

## Materials and methods

### Participants

Fourteen female soccer players (U12) (FSP) competing in a regional league with 2 years of training and twelve untrained girls (UNT) volunteered to participate in the study. The sample size was estimated using G*Power software (version 3.1) The minimum sample size to yield a statistical power of at least 0.8 with an alpha of 0.05 and a large effect size (f = 0.8) is 12. The participants were free of any known neuromusculoskeletal impairments and lower extremity injuries within the previous year. The participants were instructed to maintain their habitual lifestyle and normal dietary intake before and during the study. The physical characteristics of participants can be seen in Table 1.

**Table 1:** Physical characteristics of participants.

Variable	FSP pre (n = 14)	FSP post (n = 14)	UNT pre (n = 12)	UNT post (n = 12)
Age [yr]	11.6 ± 0.64		11.6 ± 0.49	
Height [cm]	152.7 ± 9.3	154.2 ± 9.3	150.3 ± 3.2	151.4 ± 2.8
Weight [kg]	45.0 ± 8.5	46.6 ± 8.5	41.58 ± 5.9	42.58 ± 6.2
BMI [kg/m^2^]	19.1 ± 1.8	19.4 ± 1.4	18.3 ± 1.9	18.5 ± 2.1

Data are presented as mean ± SD. *FSP* female soccer players, *UNT* untrained girls, *BMI* body mass index.

To estimate maturity status, the predicted age at peak height velocity (PHV) was calculated using the Moore equation (R^2^ = 0.91, SEE_(standard error of the estimation)_ = 0.50), previously validated^29,30^. Years from PHV were calculated for each subject by subtracting the age at PHV from the chronological age. All subjects were circa-PHV (≤ ± 1 years). None of the participants had reached menarche.

### Ethical approval

The study was conducted following the ethical aspects of the Declaration of Helsinki and was approved by the local University Senate Research Ethics Commission (24.02.2010) of the Wroclaw University of Health and Sport Sciences. The participants, coach, and parents were informed of the purposes, procedures, and risks of the tests. Prior to commencing the study, informed consent was given by all of the participants and their parents.

### Study design

**Figure 1:**
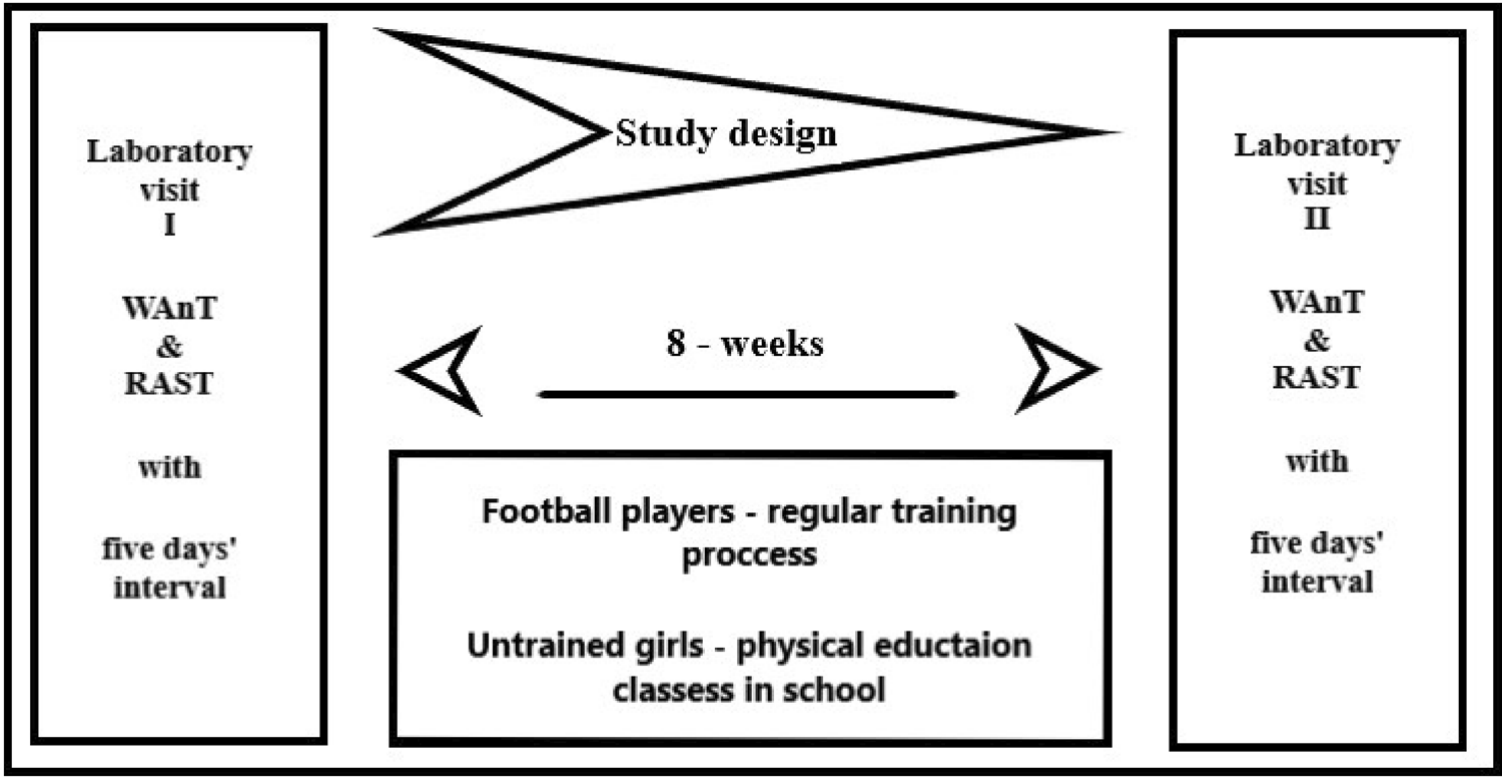
Study design.

The intervention was a non-randomised controlled before-and-after trial. Wingate anaerobic test (WAnT) and Running-based Anaerobic Sprint Test (RAST) were used in an 8-week interval to estimate the anaerobic fitness level and its changes resulting from the training process in athletes and physical education classes in untrained individuals. The WAnT was conducted on a cycle ergometer under controlled laboratory conditions and the RAST was performed on a grass turf training field (Fig. 1). The examinations were separated by 4–5 days and were performed at the same time of day (time of training)^31^. The familiarisation session for the WAnT was scheduled 1 week prior to the assessment. During the visits in laboratory the physical characteristic measurements were taken from each participant. The data were then used for WAnT load and power in RAST calculations. FSP attended three 90-min training sessions per week and a match every 2 weeks. During the training sessions, the coaches mainly focused on developing aerobic and anaerobic fitness and technical-tactical skills. Each training started with a 15′ warm-up, then 10′ of exercise developing speed followed by usual technical and tactical soccer drills training programme, small-sided games (Monday 3 × 3, Wednesday 4 × 4, Friday 3 × 3; the pitch area 5 × 10 m and 10 × 15 m; 15′–18′ duration). The intervention was carried out during the pre-season period. The team coach designed the training program. During that time, FSP also participated in physical education classes (4 times a week) in school as per the school schedule. UNT girls did not performed any additional organized activity beyond the regular physical education classes 4 times a week resulting from the school curriculum, which was confirmed by the parents. Neither the coach nor the researcher interfered with the physical education lesson program.

### Wingate anaerobic tests (WAnT)

The WAnT test was performed on a mechanically braked cycle ergometer Monark 895E (Monark, Vansbro, Sweden) with braking force of 7.5% of the participant’s body mass, which was originally proposed for children^32^. The seat height was adjusted individually so that the deflection angle was no greater than 5° when the leg was fully extended. Prior to the test, each participant performed a standardised warm-up on a Monark 895E cycle ergometer for 5 min against a workload of 50 W with two all-out sprints of 5 s each in the last minute. After 3 min of passive recovery, they completed the 30-s test. Each participant was instructed to pedal as fast as possible from the beginning and to maintain maximum speed for 30 s; verbal encouragement was given by the researchers. A resistance was applied after an acceleration phase lasting 5 s. Pedal revolution, and consequently power peak, were measured by Monark Anaerobic Test software (ver.3.3.0.0). After the test was concluded, the participants cycled slowly without resistance for the next 3 min.

The instantaneous power was averaged over 5-s intervals. The parameters were as follows: maximal power output (peak power) in absolute (PP [W]) and relative (RP [W/kg]) values; minimum power (Pmin [W]), defined as the highest and lowest mechanical powers, taken as an average over any 5-s period; mean power (MP [W]), calculated as an average power maintained throughout six 5-s segments; relative work (RW [J/kg]).

The Wingate tests were performed in controlled laboratory conditions (temperature and humidity controlled) at the Exercise Laboratory of the Wroclaw University of Health and Sport Sciences (PN-EN ISO 9001:2001 certified).

### Running-based anaerobic sprint test (RAST)

The RAST involved six 35-m maximal sprints with 10 s of recovery time between each trial. The players performed repeated sprints in alternating directions. A Smart Speed telemetric chronometer (Fusion, Australia) was used to determine each sprint interval. The test was performed on a grass turf pitch in outdoor conditions. Prior to the start of the test, the players underwent a warm-up for approximately 10 min conducted by the coaches, including a 5-min run at a self-selected pace, a few stretching exercises, and a few sprints of 10–15 m.

The variables pertaining to the sprints were as follows: peak power (PP [W]), the highest value of the six sprints; minimum power (Pmin [W]), the lowest value; mean power (MP [W]), calculated from six measured values; relative power to body mass (RP [W/kg]); relative work (RW [J/kg]). The power output for each trial was determined using the following formula: $power = weight \, \times distance^{2} \div time^{3}$^24^.

Both tests in both groups were monitored by coach responsible for training program in FSP and the same researcher.

### Statistical analysis

The software used for statistical analysis was Statistica 13 (StatSoft Inc., Tulsa, OK, USA). All results are expressed as mean ± standard deviation (SD). Data normality was assessed through the Shapiro–Wilk test. Once the assumption of normality was confirmed, parametric tests were involved. Student’s paired *t*-test was performed to assess the differences between data collected in pre and post training periods for the WAnT and RAST parameters. Cohen’s d effect size (d) was used to determine the effect size in pairwise comparisons. The magnitude of effect size was considered as trivial d < 0.2; small 0.2 < |d| < 0.5; moderate 0.5 < |d| < 0.8 and large |d| ≥ 0.8^33^. The analysis of covariance ANCOVA with baseline value as covariate was performed to examine the intergroup differences. Pearson’s correlation coefficient was calculated to establish the relationship between the power variables from the WAnT and RAST. The scale modified by Hopkins et al.^33^ was used to interpret the correlation coefficient: 0.1–0.29 = trivial, 0.30–0.49 = moderate, 0.50–0.69 = strong, 0.70–0.89 = very strong, 0.90–0.99 = nearly perfect, and 1 = perfect. Statistical significance was set at p < 0.05.

## Results

The pre-training and post-training data are presented in Table 2. The paired *t*-test shows that 8 weeks of training significantly improved anaerobic performance of examined female soccer players (p < 0.005). In UNT girls significant improvement was noticed mainly in power parameters of the RAST (p < 0.05).

**Table 2:** Wingate anaerobic test (WAnT) and running-based anaerobic sprint test (RAST) results (mean ± SD) and results of ANCOVA with baseline value as covariate.

Variable	FSP (n = 14)			UNT (n = 12)			ANCOVA
	Pre	Post	ES	Pre	Post	ES	*F*
							*p*
WAnT							
PP [W]	376.12 ± 70.12	409.90 ± 80.58^‡^	− 0.447	282.42 ± 69.24	290.32 ± 67.59	− 0.115	8.821
							0.007*
RP [W/kg]	8.39 ± 0.52	8.87 ± 0.54^†^	− 0.906	7.02 ± 1.53	7.02 ± 1.24	0	1.825
							0.184
MP [W]	326.02 ± 59.60	348.38 ± 68.23^‡^	− 0.346	239.68 ± 30.48	252.83 ± 27.15	− 0.456	7.85
							0.010*
Pmin [W]	252.86 ± 54.08	272.00 ± 56.12	− 0.347	186.57 ± 43.55	209.08 ± 47.39*	− 0.495	0.151
							0.699
RW [J/kg]	207.76 ± 13.02	213.35 ± 15.01*	− 0.398	176.44 ± 35.26	180.70 ± 30.68	− 0.129	6.315
							0.019*
TPP [s]	8.54 ± 1.36	7.77 ± 1.84	0.476	12.21 ± 3.19	10.11 ± 2.54	0.728	3.017
							0.239
Tm [s]	5.38 ± 1.49	4.51 ± 1.56	0.570	6.65 ± 3.44	7.21 ± 3.41	− 0.164	0.336
							0.568
RAST							
PP [W]	236.34 ± 49.79	282.91 ± 49.00^‡^	− 0.943	181.15 ± 51.90	203.73 ± 47.51*	− 0.454	0.935
							0.111
RP [W/kg]	5.34 ± 1.07	6.16 ± 1.01^†^	− 0.788	4.49 ± 0.70	4.98 ± 0.68*	− 0.710	0.733
							0.197
MP [W]	194.26 ± 38.16	230.69 ± 39.05^†^	− 0.944	153.71 ± 34.99	164.07 ± 33.47*	− 0.303	0.714
							0.232
Pmin [W]	158.44 ± 36.83	190.37 ± 41.02^†^	− 0.819	128.64 ± 24.30	132.95 ± 23.86*	− 0.179	0.933
							0.111
RW [J/kg]	173.72 ± 26.62	190.72 ± 23.98*	− 0.671	157.70 ± 10.60	162.78 ± 9.88*	− 0.496	0.621
							0.457

*PP* peak power, *RP* relative peak power, *MP* mean power, *Pmin* minimum power, *RW* relative work, *TPP* time to achieve peak power, *Tm* time of maintaining peak power, *FSP* female soccer players, *UNT* untrained girls, ES- Cohen’s d effect size, statistical significance: *p < 0.05; ^†^*p* < 0.005, ^‡^*p* < 0.001.

The correlation analysis between WAnT and RAST parameters shows the strong, statistically significant (p < 0.05) correlation coefficients (0.50 < r < 0.70) for pre-training and post-training power parameters (PP, Pmin and MP) in FSP, and for pre 8-week period examination for RW and post 8-week period for RP in UNT group (Table 3). In spite of strong significant and positive correlation, significantly lower rates from RAST were observed (Table 2).

**Table 3:** Correlation coefficient between WAnT and RAST parameters.

WAnT-RAST	PP [W]		RP [W/kg]		MP W]		Pmin [W]		RW [W/kg]	
	r	p	r	p	r	p	r	p	r	p
FSP pre	**0.62**	0.02	**0.62**	0.02	**0.65**	0.01	0.49	0.08	0.47	0.09
FSP post	**0.67**	0.01	**0.53**	0.05	**0.56**	0.04	0.37	0.22	0.41	0.15
UNT pre	0.31	0.33	0.13	0.68	0.26	0.41	− 0.31	0.33	− **0.58**	0.05
UNT post	0.38	0.25	0.52	0.09	0.52	0.09	− **0.62**	0.05	− 0.32	0.33

*WAnT* Wingate test, *RAST* running-based anaerobic sprint test, *PP* peak power, *RP* relative peak power, *MP* mean power, *Pmin* minimum power, *RW* relative work, *FSP* female soccer players, *UNT* untrained girls; in bold statistically significant correlation; p < 0.05.

**Figure 2:**
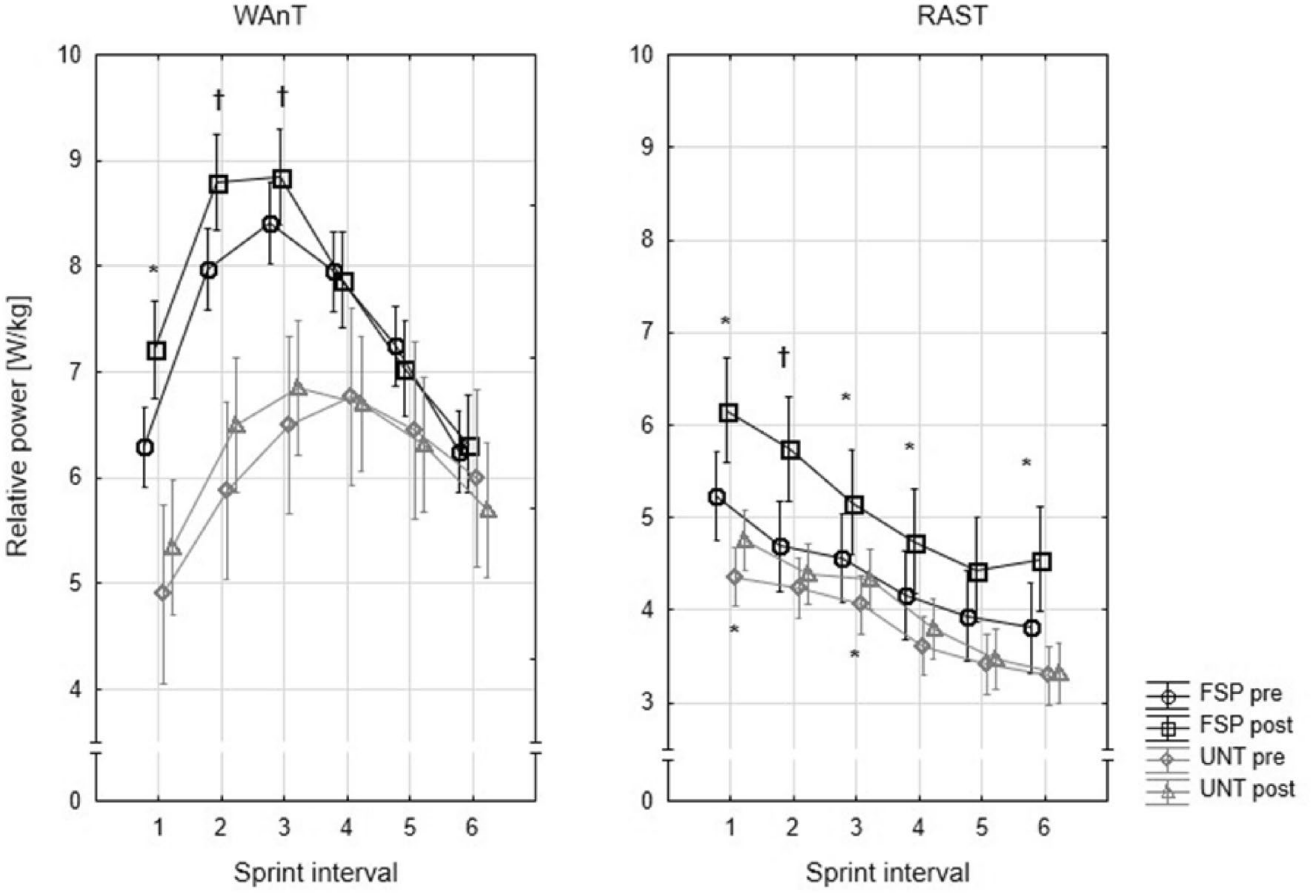
Relative power output for WAnT (each 5-s of WAnT duration) and RAST (each completed of six sprints) during the I and II examination. Significant difference between repeated tests *p < 0.05, ^†^p < 0.005.

Significant statistical differences between the two trials of WAnT and RAST in both groups are shown at Fig. 2.

The significant strong correlation for PP (1: r = 0.64, p < 0.02; 3: r = 0.71, p < 0.01; 4: r = 0.73, p < 0.01; 5: r = 0.65, p < 0.02) and for RP (3: r = 0.71, p < 0,01; 4: r = 0.76, p < 0.004; 5: r = 0.59, p < 0.04) were noted mainly for data measured in pre training conditions. In post-training conditions significant and strong correlations were noticed in interval 4 for PP (4: r = 0.63, p < 0.02) and in interval 5 for RP (r = 0.62, p < 0.02). In UNT girls the only significant strong (3: r = 0.63, p < 0.02) and very strong (1: r = 0.72, p < 0.01; 2: r = 0.81, p < 0.001) correlation were noted for PP.

## Discussion

The purpose of the study was to evaluate the relationship between the performance variables obtained during the RAST and the WAnT in female U12 soccer players and to assess the usefulness of the RAST in evaluating anaerobic performance in young girls. The main finding of this research is that the RAST can be used in place of the WAnT to evaluate the anaerobic power of young female, despite the differences in power values obtained in the two tests. It was also established that an 8-week specific soccer training significantly targeted the anaerobic performance of the young soccer player girls which can be seen in the improved results of both applied tests. An additional value of the conducted research is the confirmation of the usefulness of the RAST test in monitoring the level of anaerobic capacity in untrained girls.

The results showed significant intergroup differences in power parameters of WAnT and RAST measured before and after the 8-weeks period conditions, what indices that activity level in childhood has an impact on physical fitness. Those differences may be related to the improvement of anaerobic metabolism, increased substrates utilization, and enhanced glycolytic activity in young trained individuals^34–36^. Presented herein results also indicate that even 8-weeks of soccer training may significantly develop anaerobic capacity in youth. In FSP the improvement of power parameters in WAnT (p < 0.001) and RW (p < 0.05) were noticed, while in untrained group the only significant improvement was noticed for Pmin (p < 0.05). In the RAST in both groups were noticed significant improvement however in FSP group the development of anaerobic capacity is more significant than in untrained girls. In general, physical fitness development, and anaerobic fitness in it, is strongly related to the genetics^37^, activity level^38^, fat-free mass^19^, timing and tempo of maturity^20,21,31^. In pre-pubertal children the improvement of performance is mainly due to its activity. It dynamically start to change with maturation period even in untrained children^19^, however, the tempo and the magnitude of anaerobic capacity improvement is better in trained individuals^15,20^. The recorded increase in the values of measured parameters was twice as high as in untrained group. These results suggest that sport training in children, in general, positively affects their anaerobic capacity.

The relative power obtained in WAnT by examined girls both FSP (8.41–8.91 W/kg) and UNT (7.02 W/kg) is higher than reported by Falgairette et al.^36^ in swimmers (5.8 ± 1.0 W/kg), both active (6.3 ± 1.7 W/kg) and non-active (5.0 ± 1.1 W/kg) prepubescent boys. The differences between FSP and swimmers and UNT girls may be explained by their training experience and the specific training they have received. Similar power values were recorded in prepubescent male footballers and judokas (~ 8 W/kg)^39^, which supports the statement that practicing football may improve anaerobic performance. The results are also consisted with those obtained by Bencke et al.^40^ in female handball players (8.7 and 8.1 W/kg for elite and non-elite respectively).

Both tests used in the research showed an improvement in anaerobic performance (Fig. 2) in female soccer players U12. However, the results from WAnT suggest a greater improvement in the glycolytic system than phosphagen metabolism^41^, while RAST results could indicated an improvement in both anaerobic aspects^34^. PP and MP as measured by the WAnT improved by 8.24% (reported range for children: 4–20%) and 6.41% (reported range for children: 3–10%) respectively^23,42–44^. However, the time to reach PP is far beyond the range (3–5 s) for this parameter. In the present study the FSP needed 8.68 ± 1.65 s in the first examination and 7.91 ± 1.79 s during the examination after the 8-week training programme which is 2–3 s faster than in UNT girls. Presumably, this is due to the involvement of the glycolytic system to a greater extent than ATP-CP—which is not yet fully developed in children^35^—and to less neuromuscular coordination^45^. The MP from Wingate test correlates with the power of the anaerobic glycolysis, so an increase in MP by 6.3% indicates an improvement in the glycolytic abilities of examined FSP. Even more visible increase in power values was noted in the RAST test. Both PP and Pmin increased by 16.9% and 16.5%, respectively. In addition, the average power in RAST increased more than in WAnT (18.9% vs. 6.3%). These results suggest that RAST more precisely determines changes in anaerobic abilities in children who play soccer than the WanT. This may be due to the movement structure in the RAST, which coincides with the natural movement dominant in children's activity but also in the soccer game. This thesis is also confirmed by the results of untrained girls where improvement by 10% for PP and 6.4% for RW in the RAST were noticed. However, there is a lack of knowledge about the mechanism responsible for improving anaerobic efficiency in children^43^.

The RAST is an adopted form of the WAnT designed for running and requires a similar amount of time: ≈ 33 s in 16-year-old boys^15,23^ and 32 s in 17–19-year-old males in the RAST^23,42^ vs 30 s in the WAnT. However, the format differs significantly between the tests. The first difference is the nature of the load: in the WAnT the subject counteracts the external load imposed on the ergometer and matched to their body weight, while in the RAST the subject has to move their body weight six times on their own legs over a distance of 35 m with 10 s between runs. The second difference is the way the test is performed: the WAnT is an all-out continuous test, whilst the RAST follows an intermittent format consisting of consecutive short sprint exercises interspersed with 10-s recovery periods. These differences in the way of executing the tests translate into the power achieved on both tests, where the peak power on the WAnT is always higher than the peak power on the RAST. Presented herein results revealed significant differences between the power parameters of the RAST and the WAnT (Table 2), which is in line with other researchers’ studies^24,25,45,46^.

A significant difference was observed between the total time of six sprints in this study compared to studies in which males aged 16–19 years were examined^23,24,42^. FSP needed 39.89 ± 2.52 s and 38.21 ± 2.71 s (p < 0.01) in next examinations and UNT girls needed 41.23 ± 1.25 s and 40.71 ± 1.14 s (p < 0.06) to cover six 35-m sprints. The much longer time needed by examined herein U12 girls may be attributed to their age, reduced neuromuscular coordination or lower muscle mass^43,45^. However, herein results are consisted with those presented by Müller et al.^47^, where the untrained 14 years old girls were selected to the rugby team and subjected to rugby training for a period of 16 weeks. Their total time of six sprinting was 41.29 ± 2.83 before, and 39.59 ± 1.68 after the intervention. The results discussed here indicate that age and gender in the adolescence period have an impact on the total time. The other factor which could influence the own results is a grass surface on which the examination were conducted^48,49^. The differences in sprinting time between grass vs an artificial surface are attributed to the inefficiency of the stretch-shortening cycle and a higher contribution of glycolytic metabolism on a less rigid surface^49^.

The present study also indicated that the RAST can be used in place of the WAnT to evaluate the anaerobic power of young female soccer players, despite the differences in power values obtained in the two tests. A strong and significant correlation was found between the RAST and WAnT power parameters—PP, Pmin, and MP (0.50 < r < 0.69)—for both pre- and post-training measurements. The results correspond with correlation values for peak and mean power reported by Burgess et al.^26^ in a study performed on adult male amateur football players. Higher correlations between the RAST and the WAnT for peak and mean power (r = 0.82 and r = 0.72, respectively) were found in a comparative study conducted on male soldiers^22^ or on young male soccer players aged 14–17 years (r = 0.81 and r = 0.86, respectively)^25,42^. The results presented in this article confirm the correlations between WAnT and RAST parameters in trained subjects. There were strong correlations between PP, Pmin (r = 0.62), and MP (r = 0.65) in absolute values for pre-training conditions, as well as PP (r = 0.67), Pmin (r = 0.53), and MP (r = 0.56) for post-training conditions. The incomplete correlation reached in herein research partially may be explained by different movement patterns and activated muscle mass. In the WAnT the limited muscle groups are engaged against the external load, whereas in RAST a greater muscle mass in engaged while running. Metabolic processes and resulting neuro-mechanical changes during both test executions may also be the cause^25,34^. Furthermore, the neuromuscular coordination necessary to perform RAST may be also essential, and in prepubertal age is still underdeveloped^15,19^. So far, comparative studies of the RAST and WAnT have focused on male athletes including soccer players^22,25,26,45^. To the best of the author’s knowledge, no comparative study of the RAST and the WAnT in female U12 soccer players has been published to date. The lack of significant correlations in untrained group between RAST and WAnT parameters may be attributed to the lower coordination level and anaerobic metabolism comparing to trained soccer players girls^35,43^.

The advantage of the RAST is its simple procedure (35 m of running with 10 s of rest, which may seem ideal for assessing the repeated sprint ability (RSA) in athletes of field-based team sports). Because the RAST can be used to measure power^22,24,46,50^ it seems to be a suitable way to assess anaerobic performance against the very popular and traditionally used WanT. The Wingate test, with its benefits, has one drawback to running sports: it is an ergometric test with a movement structure that does not naturally occur whilst running, and additionally, it can be too heavy for untrained children. By contrast, the RAST is based on running, which is more suitable for untrained and athletes of sports where running is the basis of movement. The nature of soccer game, with its exposure to repeated sprints of short duration (2–7 s) with only a brief recovery time, should promote tests based on similar movement behaviour in assessing anaerobic capacity. The RAST has a strong association with maximal speed^51^. The nature of RAST also allows for a more frequent assessment of the progress of players, even as young as U12, and the lack of stress associated with visiting the laboratory is an additional benefit for it. Furthermore, it can be easily added to the training routines of soccer players or other team games e.g. rugby^47^. The disadvantage of RAST is the lack of multidirectional running which is typical of the game of football and forms the basis for the RSA tests. Regardless, the RAST is a quantitative measure and not just a qualitative measure, as with the RSA tests, which increases its usefulness in soccer training. Finally, it can be easily performed by untrained children because it is based on natural movement of running and such as is performed without any additional external load.

The main limitations of the present study are its small sample size. Unfortunately, we did not have access to a larger group of female soccer players. The tiny female soccer players group did not allow the division into two subgroups in relation to the training carried out. Also, the completing control group wasn’t an easy. Enrolling untrained children in this experiment seemed unethical, as children aged 11–12 years are not prepared for such a strenuous effort as the WAnT. Nevertheless, we got permission from 16 parents for competing the tests, however, after completing of the first WAnT, four guardians withdrew their consent.

## Conclusions

This is the first study to examine the relationship between power data collected in the WAnT and the RAST in U12 girls. The observed improvement of anaerobic performance, in response to training applied, accompanied by the strong correlation noted between the WAnT and the RAST power parameters prove the usefulness of the RAST test in assessing anaerobic capacity in young trained girls. The simplicity of this test and the similarity in the structure of movements to the structure of the sport being practiced also encourages its use in monitoring the training process of young female soccer players. An additional value of the conducted research is the confirmation of usefulness of the RAST in monitoring the changes in level of anaerobic capacity resulting from the development process and school physical education programme in untrained 10–11 year old girls, which is to the authors knowledge a novelty. However, the sample group was small, which is a certain limitation and reduces the possibility of generalizing the obtained results. Further studies with larger samples and other sport disciplines are needed especially in female players, as results in this area are still very scarce.

## Data Availability

The data presented in this study are available on request from the corresponding author.
